# Conservation genetics of the bonnethead shark *Sphyrna tiburo* in Bocas del Toro, Panama: Preliminary evidence of a unique stock

**DOI:** 10.1371/journal.pone.0220737

**Published:** 2019-08-15

**Authors:** Cindy Gonzalez, Austin J. Gallagher, Susana Caballero

**Affiliations:** 1 Departamento de Ciencias Biológicas, Universidad de los Andes, Laboratorio de Ecología Molecular de Vertebrados Acuáticos—LEMVA, Bogota, Colombia; 2 Beneath the Waves Inc, Herndon, Virginia, United States of America; 3 Smithsonian Tropical Research Institute, Balboa, Panama City, Republic of Panama; 4 Rosentiel School of Marine and Atmospheric Sciences, University of Miami, Miami, Florida, United States of America; Department of Agriculture, AUSTRALIA

## Abstract

The bonnethead shark, *Sphyrna tiburo*, is a small elasmobranch distributed in the Eastern Pacific from southern California to Ecuador, and along the Western Atlantic, with preferences for continental margins of North, Central and South America, the Gulf of Mexico, and the Caribbean. Recent studies have suggested that it could be under a process of cryptic speciation, with the possibility to find different species in similar geographic locations. Here we assessed the population structure and genetic diversity of this highly philopatric and non-dispersive species in the Bocas del Toro Archipelago, Panama. Fragments of the mitochondrial genes cytochrome oxidase I and control region, were used to test the genetic structure of adult and juvenile *S*. *tiburo* in this area, and were compared with other locations of the Western Atlantic and Belize. We found significant genetic differentiation between Caribbean bonnethead sharks from Bocas del Toro and Belize, when compared to bonnetheads from other locations of the Western Atlantic. These results also suggest that Bocas del Toro could constitute a different genetic population unit for this species, whereby bonnethead sharks in this area could belong to a unique stock. The information obtained in this study could improve our understanding of the population dynamics of the bonnethead shark throughout its distribution range, and may be used as a baseline for future conservation initiatives for coastal sharks in Central America, a poorly studied an often overlooked region for shark conservation and research.

## Introduction

Molecular approaches can provide powerful tools for augmenting our understanding of the population features, connectivity, and conservation needs of highly dispersed, mobile marine species, such as sharks [1–4]. The understanding of genetically distinct populations is especially important to identify and clarify evolutionary processes such as cryptic speciation, that refers to species complexes that have been classified as a single species, are phenotypically or morphologically similar, but genetically different, [5,6]. As a consequence of overfishing and due to their low rates of biological productivity (slow growth, late maturity, low fecundity), population declines have been reported for many species of sharks in the last several decades, emphasizing the need to increase our understanding of their distribution ranges and life histories [7–11], in order to implement appropriate conservation measures. Over the past two decades, genetic studies have become increasingly informative for refining and informing management strategies in light of the growing threats to sharks worldwide [12].

Hammerhead sharks are members of the family Sphyrnidae, which is composed of 11 species, of which 10 belong to the genus *Sphyrna* and one to the genus *Eusphyra* [9]. From the *Sphyrna* genus, the bonnethead shark *Sphyrna tiburo* (L.1758) is one of the five small-bodied species (*<*150 cm total length at maturity), whose biological processes are different than those of the larger species (e.g. *Sphyrna lewini*) [13]. *Sphyrna tiburo* is distributed in the Eastern Pacific from California to Ecuador, and throughout the Western Atlantic from North Carolina (USA) to southern Brazil, including the Gulf of Mexico and the Caribbean [14,15]. This species is considered relatively abundant and is a common seasonal resident among insular and estuarine waters, which serve as critical habitat for feeding, mating, gestation and parturition [13,16–18].

Hammerhead sharks are known to migrate long distances where mating and parturition areas are often separated [18,19], thereby promoting geographical and genetic connectivity. However, bonnethead sharks do not appear to migrate long distances. Instead, individuals (juveniles and adults) return to areas in close proximity to the sites where they were born, exhibiting high site fidelity and philopatry, which could be related to reproduction, mating behavior, and availability of food sources [20–22]. Differences in life history traits (e.g. variation in reproduction, size at birth, growth rates, size and age at maturation) among similar geographic locations throughout the US Western Atlantic have been found for this species [17,23], suggesting that environmental factors, latitudinal variation, and resource availability play key roles in the evolutionary processes of different bonnethead shark populations [23,24]. *S*. *tiburo* is considered highly productive due to its rapid growth rate, short gestation period and high biological productivity [17,20,23], rendering it less susceptible to exploitation when compared to other larger congeneric species (*S*. *lewini*, *S*. *mokarran*, *S*. *zygaena)* [17,18,25]. Hence, has been assessed as “Least Concern” by the International Union for Conservation of Nature (IUCN) [7]. However, as fishery data is largely absent for this species in the Caribbean, the level of exploitation and its impact on the populations are unknown [26].

Taxonomically, *S*. *tiburo* is considered to be a widely-distributed species, composed of a single panmictic population in the Western Atlantic [13,27]. However, strong site fidelity could result in closed populations that are genetically different, while morphologically identical. Therefore, bonnethead shark populations could be experiencing cryptic speciation [6,13,20,27].

Previously, Naylor et al. [28] found large genetic divergence by analyzing and comparing a fragment of the mitochondrial *ND2* gene, from twelve bonnethead sharks from the Gulf of Mexico, and two individuals from Trinidad. It was suggested that the animals from the two locations belong to two different species (the Trinidad specimens were designated as *S*. *cf*. *tiburo*). Additionally, Escatel-Luna et al. [27] found significant population structure between 251 bonnethead sharks from neighboring estuaries in the U.S. Western North Atlantic and the Gulf of Mexico, suggesting that there are multiple populations within this well-studied region. Subsequently, Fields et al. [13] provided strong evidence of significant population structure and cryptic speciation in bonnethead sharks from the Western Atlantic (n = 181) and Belize (n = 58), by analyzing fragments of the mitochondrial CR, COI, and *ITS-2* genes, and maintained the two species (*S*. *tiburo* and *S*. *cf*. *tiburo)* proposed by Naylor et al. [28] remarking that they should be managed independently.

An improved understanding of genetic population structure and clarification of taxonomic differences between populations of *S*. *tiburo*, may add important geographical resolution, since *S*. *tiburo* is commercially important in the U.S., Mexico, Brazil, Belize, Panama, and the Caribbean [7,29,30]. This is essential for the elucidation of potential cryptic species, and to ensure that the different stocks (population units) can be harvested sustainably [6,13,21,26].

The primary aim of this study was to investigate the genetic diversity and population structure of *S*. *tiburo* in Bocas del Toro (BDT), Panama, by analyzing fragments of the mitochondrial genes cytochrome oxidase I (COI) and control region (CR). We confirmed species identity by barcoding and subsequently compared the obtained sequences with similar data available from Belize (BZ), the Western Atlantic (WA) and the Gulf of Mexico [13,31–33]. Finally, we discuss our results as they relate to the potential of the existence of an independent stock of *S*.*cf*. *tiburo* in BDT, and subsequently, for management implications [1,13,21].

## Materials and methods

### Study area

**Fig 1:**
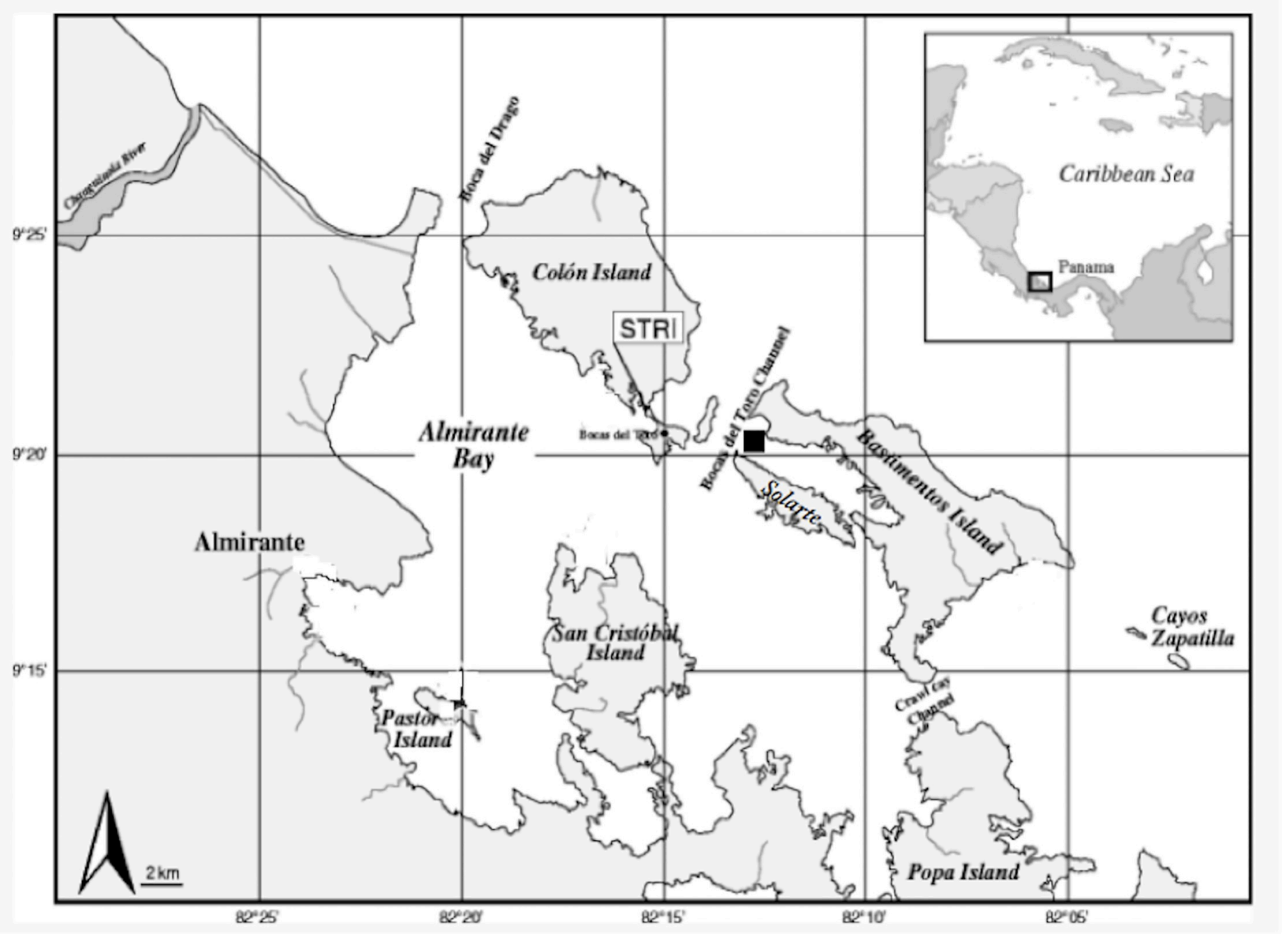
Map of the Bocas del Toro Archipelago. The Black box indicates the sampling site that corresponds to Hospital Point in Solarte Island. Modified from Seemann et al. (2014).

The BDT archipelago is located in the northwest of the Republic of Panama, between 8°30' and 9°40'N and between 82°56' and 81°8'W. This area in the Caribbean Sea, comprises a continental shelf, seven large islands, and other smaller islands fringed by mangrove cays [34–36]. This study was conducted off Solarte Island (Fig 1), an inshore coastal area surrounded by mangroves, coral reefs and shallow seagrass beds. These areas meet the criteria for nursery habitats for small coastal sharks [22].

### Sampling and DNA extraction

Samples were collected between October 2016 and January 2017. Fifteen bonnethead sharks were captured at Hospital Point (Fig 1) during nighttime, using artisanal rod and reel techniques. The total length (TL) and sex were recorded, and a fin clip was sampled from each shark. Individuals were either juveniles or adults (TL: 49–106 cm) and were released alive after sampling. The methodology for sample collection was approved by the Smithsonian Tropical Research Institute IACUC (Institutional Animal Care and Use Committee–Record ID 20676904 and 20676903), and the Autoridad Nacional del Ambiente de Panama (ANAM) (permit number 05870- SEX/A-2-17). Fin samples were stored in 95% ethanol for genetic analyses and exported to Bogota, Colombia under the CITES permit number 05870. Total DNA extraction was performed using the protocol 9 of the Bioline ISOLATE II Genomic DNA Kit (http://www.bioline.com/us/isolate-ii-genomic-dna-kit.html).

### COI amplification and sequencing

A 639 base pair (bp) fragment of the mitochondrial COI region was amplified for all samples (n = 15), using the primers FishCoxI F1 (5´TCWAC-CAACCACAAAGAYATYGGCAC) and FishCoxI R1 (TAR-ACTTCWGGGTGRCCRAAGAATCA), modified from Ward et al. [37]. Polymerase chain reaction (PCR) was performed as follows: 94°C for 2 minutes, 35 cycles of 30s at 94°C, 55°C for 45s, and 72°C for 40s, followed by a final extension step of 72°C for 10 min. Successfully amplified PCR products were purified using Exo-SAP (Thermo Scientific) and sequenced on an ABI 3500.

### CR amplification and sequencing

A 1064 bp fragment of the mitochondrial CR was amplified for all samples (n = 15), using the primers Pro-L (5`AGGGRAAGGAGGGTCAAACT3´) and 12SrRNA (5´AAGGCTAGGACCAAACCT3´), modified from Quintanilla et al. [32]. PCR was performed for 35 cycles of 1 min at 95°C, 1 min at 61.4°C, and 2 min at 72°C, followed by a final extension step of 72°C for 10 min. Successfully amplified PCR products were purified using Exo-SAP (Thermo Scientific) and sequenced on an ABI 3500.

### Alignment and statistical analysis

**Fig 2:**
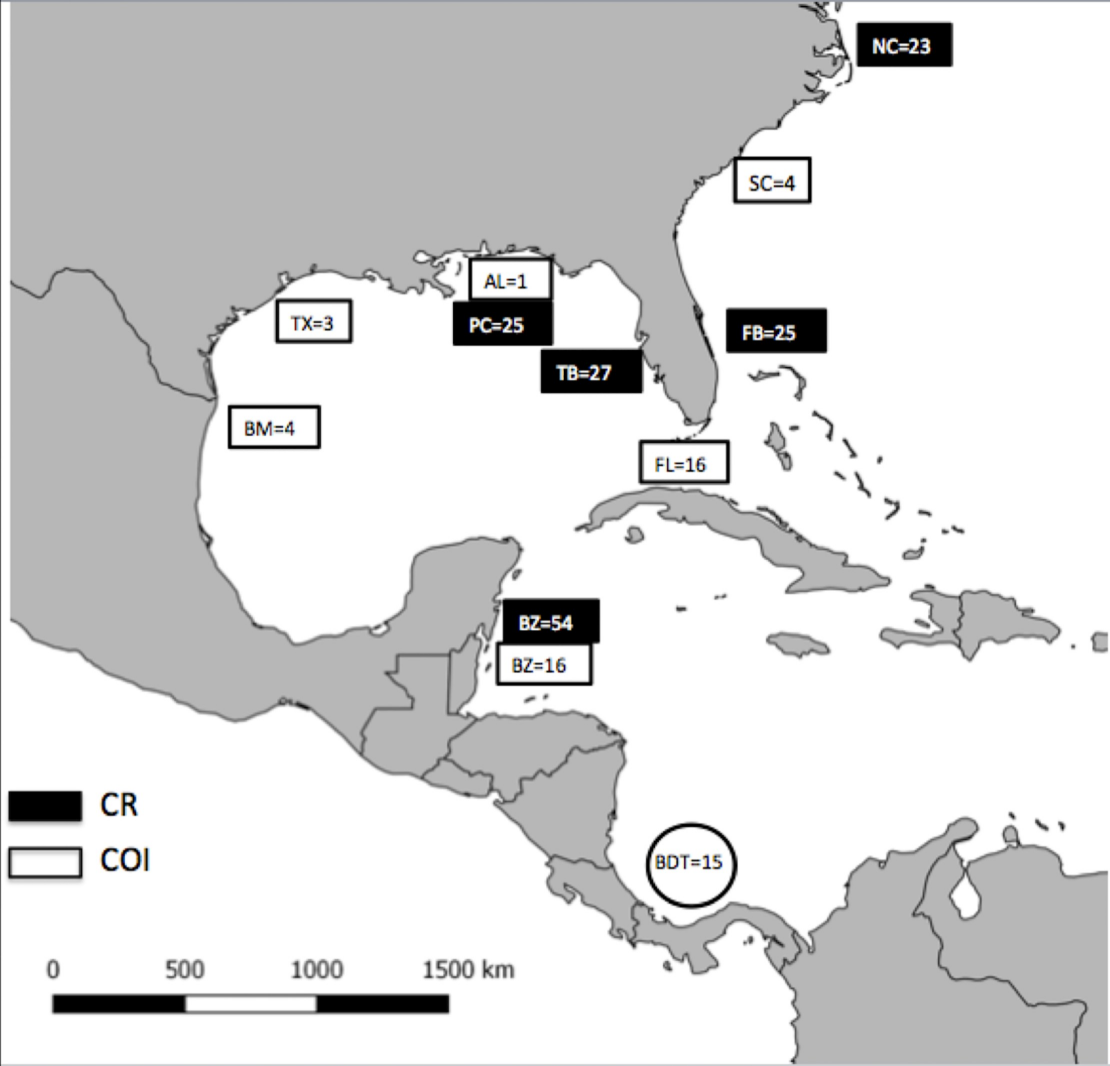
Map of localities and sampling replication. Bocas del Toro (BDT COI and CR). COI sequences: Alabama (AL), Bagdad Mexico- Gulf of Mexico (BM), Belize (BZ), Florida (FL), South Carolina (SC), Texas (TX). CR sequences: Florida Bay, Florida (FB), Tampa Bay, Florida (TB), Panama City, Florida (PC), and Belize (BZ).

All sequences were edited and checked manually using Geneious v.3.6.1 (http://www.geneious.com) and aligned using MacClade 4.08 software, which was also used to identify haplotypes [38]. An additional 44 COI sequences from other localities around the WA were obtained from GenBank and used for comparisons. These included samples from a previous study from Wong et al. [33]: Alabama (AL = 1), South Carolina (SC = 4), Belize (BZ = 16), Florida (FL = 16), Texas, Gulf of Mexico (TX = 3), and Bagdad Beach, Gulf of Mexico (BM = 4) (S1 Table). Another 44 CR haplotype sequences of *S*. *tiburo* were obtained from GenBank from the WA: North Carolina (NC = 23), and from the following locations in Florida: Tampa Bay (TB = 27), Florida Bay (FB = 25) and Panama City (PC = 25), using the data from Portnoy et al. [21] (S2 Table). Additionally 54 CR sequences from BZ, previously published by Fields et al. [13] were used for further comparisons (Fig 2).

A statistical parsimony network for each gene fragment was constructed using the software TCS v. 1.21 [39], providing a 95% plausible set for all haplotype linkages. The program JModelTest v.2.3.1 was used to obtain the best model of DNA substitution [40]. After selecting the best model (TrN+I), iTol (www.itol.embl.de) was used to build a COI maximum likelihood tree, including an outgroup *Carcharhinus leucas* (GenBank: FJ519612.1), and sequences from sister species *Sphyrna lewini* (GenBank: FJ519636.1) and *Sphyrna tudes* (GenBank: FJ519524.1). For the CR, the best model selected was HKY+i, that was used to create the maximum likelihood tree in PAUP [41]. For the CR, the following sequences were used as sister species obtained from GenBank: *Sphyrna media* (GenBank: GU385317.1) [42], and *S*. *lewini* (KY315830.1).

Genetic diversity was calculated as haplotype diversity (*h*) and nucleotide diversity (π) for each gene fragment. Population differentiation was tested via pairwise comparisons of both *F*_*ST*_ and *Φ*_*ST*_ using ARLEQUIN v.3.5.1.2 [43], with 10,000 permutations. Genetic differences among population units based on geographic locations (WA and, BZ and BDT) were quantified by an analysis of molecular variance (AMOVA) as implemented in Arlequin, using 10,000 permutations [43]. A Bonferroni correction to adjust the *P*-value was also performed to take into account the number of pairwise comparisons for the localities.

## Results

Fifteen *S*. *tibur*o samples were confirmed by DNA barcoding by amplifying a 630 bp fragment of the mitochondrial COI gene, and were compared against other sequences available in GenBank by using BLAST.

### mtDNA cytochrome oxidase I (COI)

**Fig 3:**
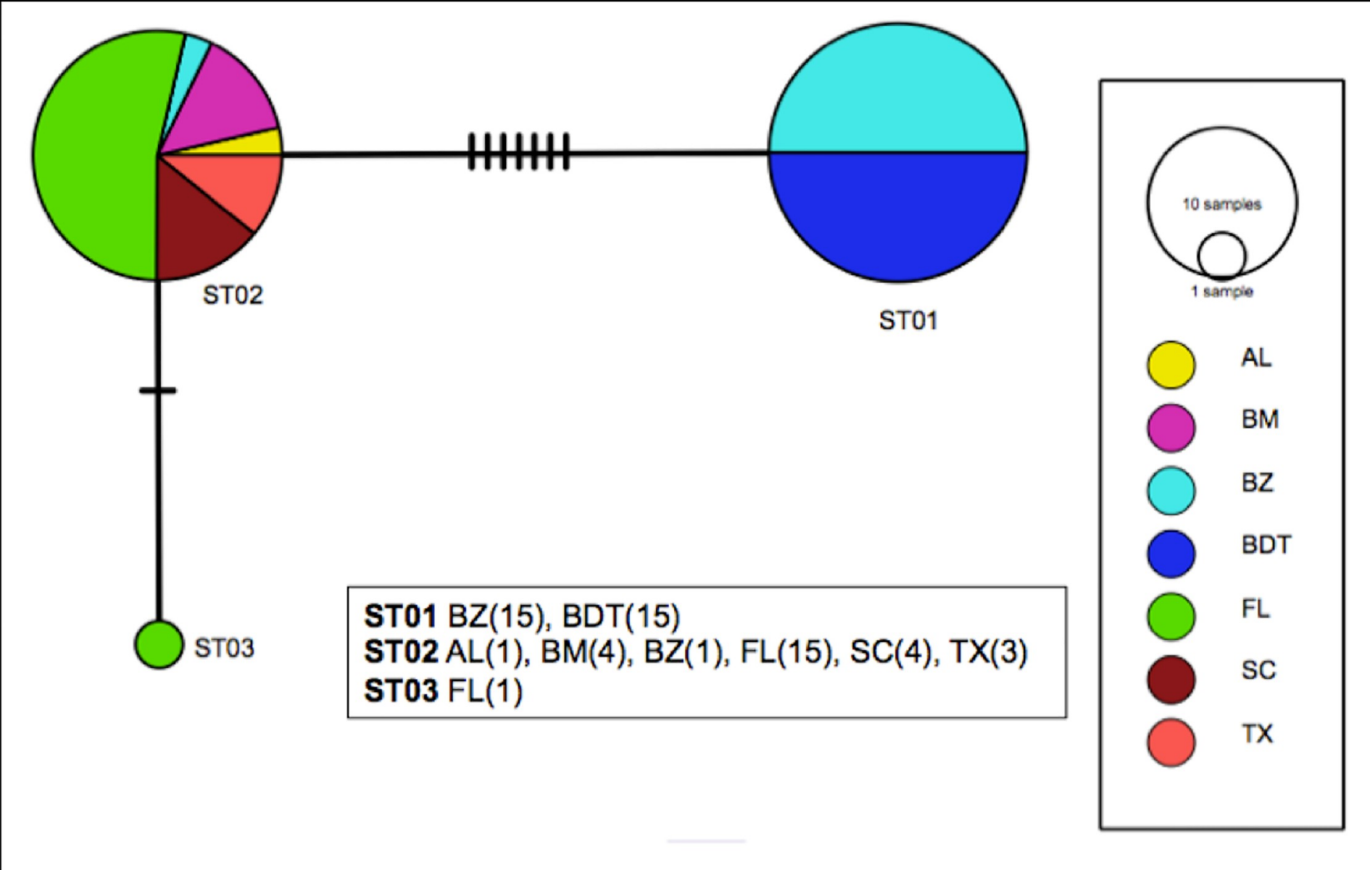
Maximum parsimony network of the cytochrome oxidase I (COI) for *S*. *tiburo*. Circles are sized proportional to haplotype frequency and color coded for location. Sample sizes are as follows: Bocas del Toro (BDT = 15) and GenBank sequences: Alabama (AL = 1), Bagdad Mexico (BM = 4), Belize (BZ = 16), Florida (FL = 16), South Carolina (SC = 4), Texas Gulf of Mexico (TX = 4).

**Fig 4:**
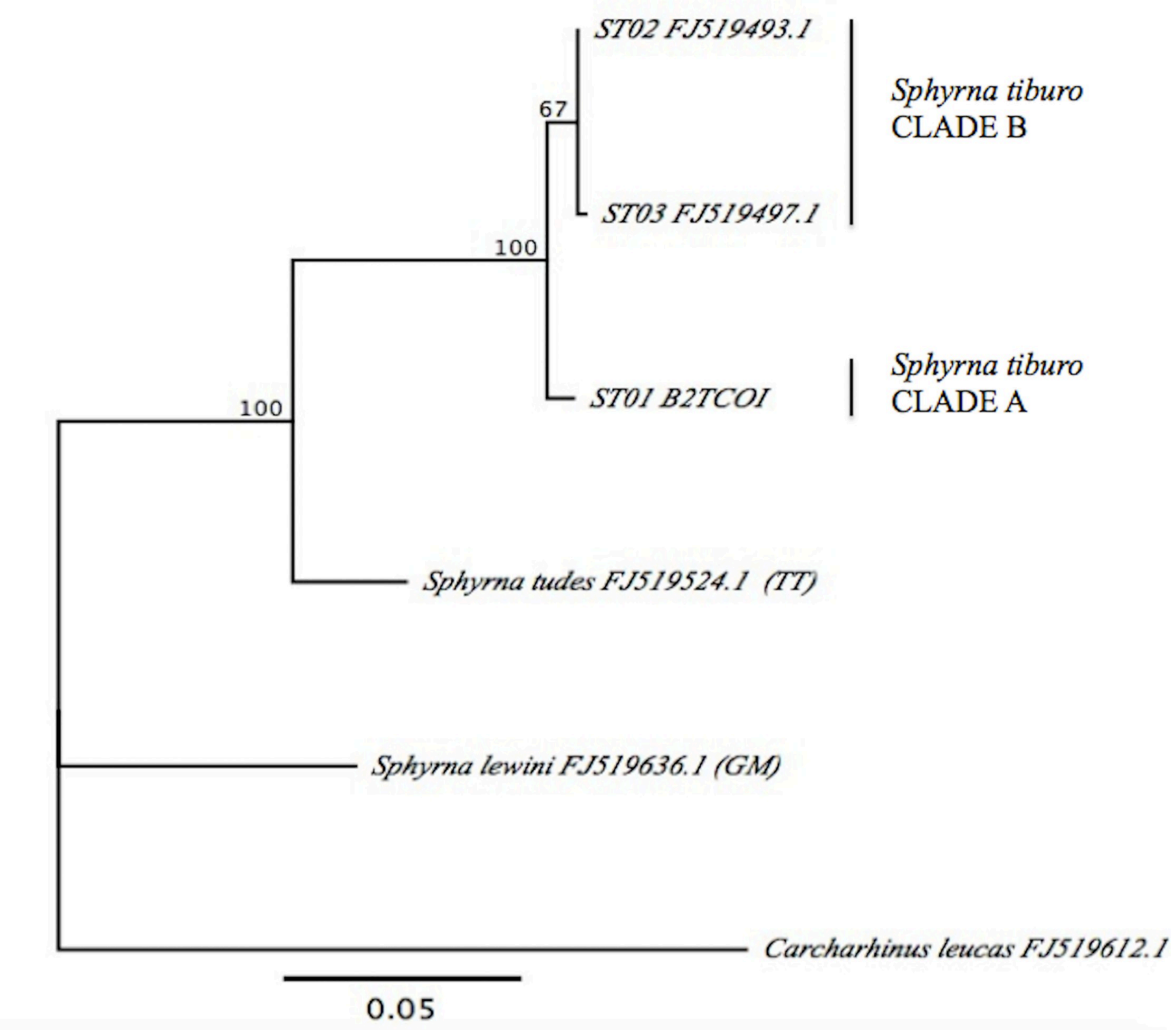
Maximum likelihood distance tree of COI haplotype sequences from *S*. *tiburo* and outgroups: *S*. *lewini*, *S*. *tudes* and *C*. *leucas*. Clade A corresponds to BDT and BZ. Clade B corresponds to the localities of the WA and the Gulf of Mexico.

A fragment of 639 bp of the COI gene was obtained from 15 samples of *S*. *tiburo* from BDT (n = 15). Another 44 COI sequences reported in GenBank from other localities of the WA and BZ (Wong et al. [33]), were included in our analyses (S1 Table). Within this combined dataset, we identified three distinct haplotypes: ST01 which included all samples from BDT and all except one sample from BZ, and was separated by seven changes from ST02; ST02, was the most common and probably the most ancestral haplotype, which included all the localities from the WA and the Gulf of Mexico, but was only found in one sample from BZ and in none of the BDT samples (Fig 3); and ST03, was a very distinct and unique haplotype from Florida. Eight variable sites were identified (Table 1). The haplotype network obtained from the TCS analysis and the phylogenetic reconstruction showed two differentiated groups or clades, separating BDT and BZ from the other localities in the Gulf of Mexico and the WA (Figs 3 and 4), meaning that these populations are reciprocally monophyletic. Overall haplotype diversity was *h =* 0.489. Nucleotide diversity (π) was low and similar for all the sites, with values ranging from 0.00% (BDT, SC, AL, BM), 0.053% for TX, and 0.1369% for BZ (Table 2).

**Table 1:** Eight variable sites over 639 bp of the mitochondrial COI gene determining 3 haplotypes for *S*. *tiburo*.

**Variable Sites**								
**Haplotypes**	169	283	322	373	412	529	532	575
ST01	T	G	T	G	G	C	G	C
ST02	C	A	C	A	A	T	G	T
ST03	C	A	C	A	A	T	A	T

**Table 2:** Pairwise *F*_*ST*_ values (above diagonal) and *Φ*_*ST*_ values (below diagonal) for the COI gene from the Atlantic and Caribbean populations of *S*. *tiburo*. Bocas del Toro (BDT), and other localities from GeneBank: Bagdad Mexico (BM), Belize (BZ), Florida (FL), South Carolina (SC) and Texas (TX).

*F*_*ST*_ Φ_ST_	BM (n = 4)	BZ (n = 16)	BDT (n = 15)	FL (n = 16)	SC (n = 4)	TX (n = 3)
**BM**	h = 0.8333 ± 0.2224 π = 0.000 ± 0.000	0.5901***	0.8091***	0.1039	0.1667	-0.0909
**BZ**	0.8923***	h = 0.2417 ± 0.1353 π = 0.0014 ± 0.0011	0.0230	0.6883***	0.7974***	0.5853***
**BDT**	1.000***	-0.0087	h = 0.00 ± 0.000 π = 0.000 ± 0.000	0.8145***	1.000***	0.8275***
**FL**	-0.1348	0.9245***	0.9905***	h = 0.3500 ± 0.1478 π = 0.0002 ± 0.0003	-0.0821	0.2288
**SC**	0.0000	0.8923***	1.000***	-0.1347	h = 0.000 ± 0.000 π = 0.000 ± 0.000	0.3514
**TX**	0.0000	0.8860***	1.000***	-0.1940	0.0000	h = 1.000 ± 0.2722 π = 0.053 ± 0.000

Significant P values at <0.005*

<0.002**

< 0.001***

Probability values based on 10,000 permutations. Significant P-values in bold. Haplotype (h) and nucleotide (π) diversity values % ± standard deviation are shown in the diagonal for each locality. Numbers of samples of each locality are shown in parentheses. Values were taken after a Bonferroni correction.

Pairwise estimates of *F*_*ST*_ and *Φ*_*ST*_ values (Table 2) revealed significant differentiation between BDT and BZ, from BM, FL, SC and TX (*F*_*ST*_ = 0.72961 *P* = 0.000+-0.000), which is also consistent with the phylogenetic tree. AL was not included in this analysis due to small sample size. In general, *Φ*_*ST*_ values showed higher values when compared to *F*_*ST*_ estimates, ranging from *Φ*_*ST =*_ 0.000 to 1.000. However, these values exhibited high variation between sites, showing significant differences for *F*_*ST*_, revealing that BDT and BZ samples were distinct from all other sites. This was also confirmed by the maximum parsimony network, and the maximum likelihood tree, which segregated these two sites apart from other locations.

The AMOVA indicated, that the genetic differentiation was significant between the combined BDT and BZ, and the other WA sites (Φ_ST_: 0.9568. P< 0.000). Pairwise *F*_*ST*_ was also significant, showing high population structure between sites (*F*_*ST*_: 0.72961 P: 0.000).

### mtDNA- control region (CR)

**Fig 5:**
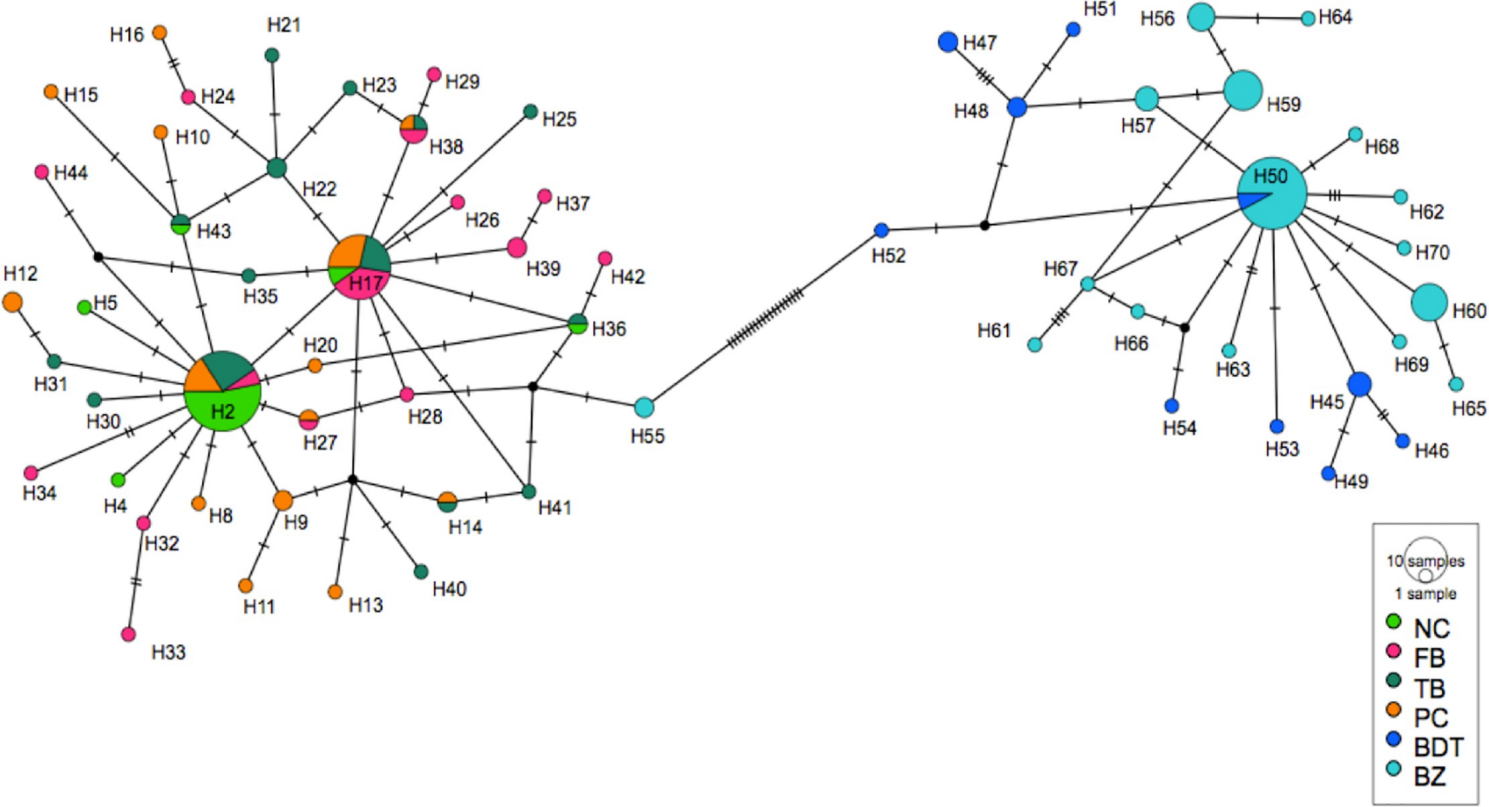
Maximum parsimony network for *S*. *tiburo* CR haplotypes. Circles are sized proportional to haplotype frequency and color coded for location. Small black dots and hatch marks along the branches represent mutational steps that were not observed in this study. Sample sizes are as follows: Bocas del Toro (BDT = 15), Belize (BZ = 54), and GenBank sequences: North Carolina (NC = 23), Florida Bay (FB = 25), Tampa Bay (TB = 27), Panama City (PC = 25). For details and GenBank accession numbers see S2 Table.

**Fig 6:**
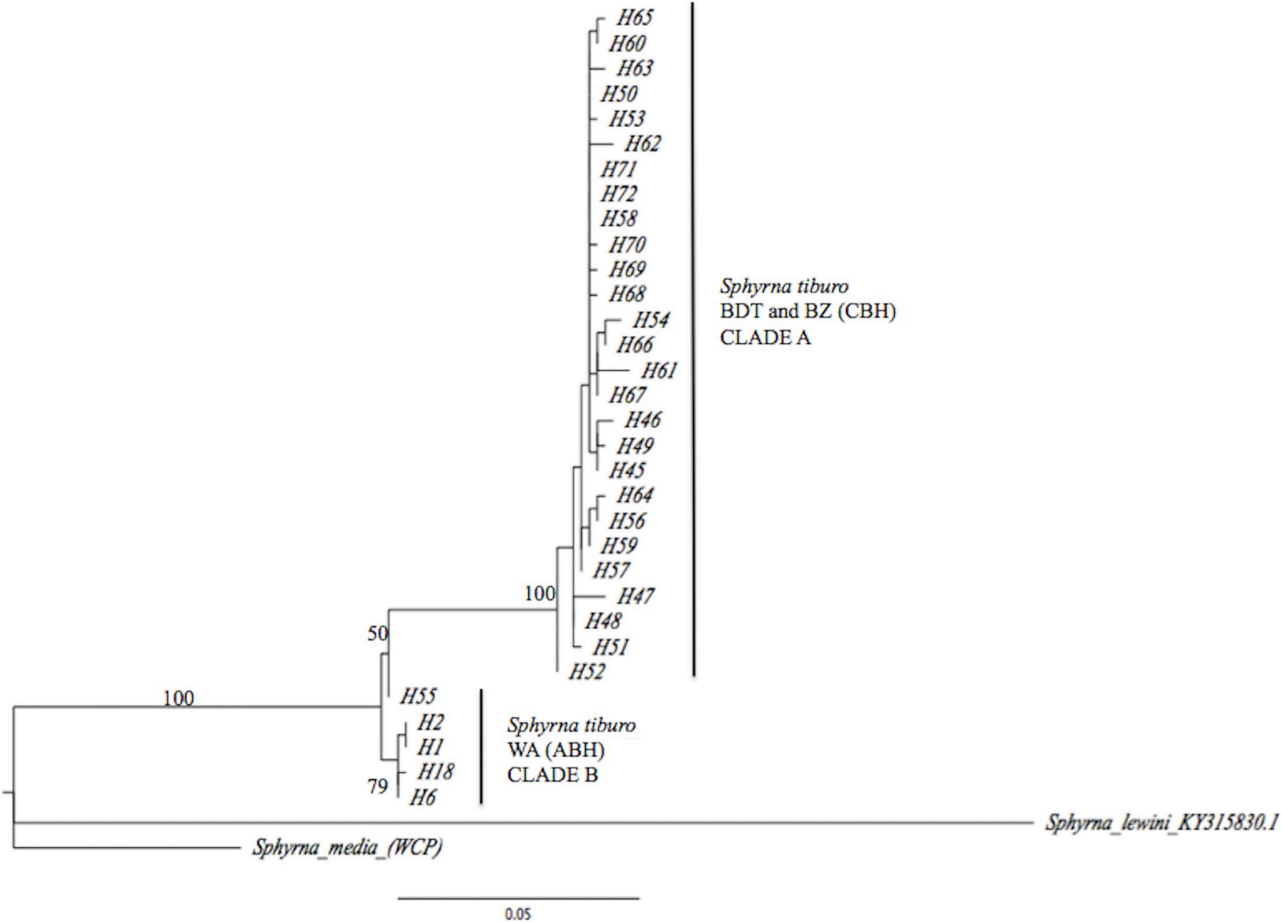
Maximum likelihood distance tree for CR haplotype sequences from *S*. *tiburo*, and outgroups: *S*. *media* (Eastern Caribbean Panama—GU385317.1), and *S*. *lewini* (WA—KY315830.1). Bocas del Toro (BDT = H45-54) and most common haplotypes from the WA from GenBank sequences from North Carolina (NC), Tampa Bay (TB), Florida Bay (FB) and Panama City (PC). ML support values are given in the branches.

A fragment of 1,064 bp of the CR gene from 15 bonnethead sharks sampled from BDT was amplified and sequenced. A total of 10 unique CR haplotypes (H45- H54), defined by 12 variable sites, were identified for BDT (Table 3); another 44 bonnethead shark CR haplotype sequences (H1-H44) from the WA (NC, TB, FB, and PC) (Wong et al. [33]), were compared to the BDT haplotypes (S2 Table). An additional 54 sequences from BZ (H55-H72) (Fields et al. [13]) were also used for further comparisons. The haplotype network obtained from TCS separated two groups, differentiated by 20 mutational steps; one grouped BDT and BZ haplotypes, and the second grouped all locations from the WA (Fig 5). Only one haplotype (H51) was shared between BDT and BZ, but no haplotypes were shared with localities from the WA, except for H55 from BZ that was placed as a WA haplotype. Both WA and Caribbean haplotypes exhibited a star-like phylogeny, which could represent a recent population expansion event from a common ancestral haplotype. For the WA, H2 was the most common and ancestral haplotype with a frequency of 21% among samples from NC, TB, FL and PC, followed by haplotype H6 with a frequency of 15%. Both the topology of the maximum parsimony network and the maximum likelihood tree (Figs 5 and 6) showed a similar pattern, with BDT and BZ appearing to be a differentiated lineage from the other WA locations. These results suggest that BDT and BZ constitute an independent evolutionary lineage differentiated from *S*. *tiburo* from the WA.

**Table 3:** Twelve variable sites over 1064 bp of the mitochondrial CR gene determining 10 haplotypes for *S*. *tiburo*.

Variable Sites												
Haplotypes	250	341	401	448	472	488	509	520	715	813	861	958
H45	G	A	T	A	C	G	G	C	A	G	A	A
H46	A	A	T	A	C	G	G	C	G	G	A	A
H47	A	A	G	A	T	G	A	C	A	A	G	G
H48	G	A	T	A	C	G	G	C	A	A	G	G
H49	G	A	T	A	C	G	G	T	A	G	A	A
H50	G	A	T	A	C	G	G	C	A	G	A	G
H51	G	T	T	C	C	G	G	C	A	A	G	G
H52	G	A	T	A	C	G	G	C	A	A	A	G
H53	G	A	T	A	C	A	G	C	A	G	A	G
H54	G	A	G	C	C	G	G	C	A	G	A	G

In general, haplotype diversity was high (*h* = 0.898) with the lowest value for NC (*h* = 0.7194), which contained six haplotypes dominated by H43, whereas other localities contained haplotypes in lower frequencies. For BDT, haplotype diversity was also high (*h* = 0.9429). Mean nucleotide diversity (π) was 0.23%, with differences between haplotypes being based on two variable sites, with values ranging from 0.10% for NC to 0.32% for BDT (Table 3).

*F*_*ST*_ and *Φ*_*ST*_ pairwise estimates showed highly significant values between most populations after Bonferroni correction (Table 4). In general, *Φ*_*ST*_ values were greater when compared to *F*_*ST*_ estimates, but both estimators showed a similar pattern with significant genetic differentiation between sharks from BDT and BZ and sharks from all WA locations (values ranging between *Φ*_*ST*_ *= 0*.*8846* and *Φ*_*ST*_ *= 0*.*9334)*. There was also no significant differentiation between several locations in the WA (i.e. FB, TB and PC), while NC showed significant differentiation from all other sites (values ranging between *Φ*_*ST*_ *=* 0.0480 and *Φ*_*ST*_ *=* 0.9254). *F*_*ST*_ values showed a similar pattern, with BDT and BZ as significantly differentiated from all WA locations (*F*_*ST*_ *=* 0.08543; P = 0.000), and NC also significantly differentiated from all other sites (*F*_*ST*_ *=* 0.0551 to *F*_*ST*_ *=* 0.1754). Population structure was also detected between BDT and BZ, suggesting two differentiated populations in this lineage (*Φ*_*ST*_ *=* 0.0912, *F*_*ST*_ *=* 0.1233; P = 0.000). A global AMOVA (Table 5) using both *F*_*ST*_ and *Φ*_*ST*_ estimates was consistent with the differences observed from pairwise sample comparisons (*Φ*_*ST*_ = 0.9097; P = 0.000, Table 5). A significant estimate for the variance component among populations within groups was also confirmed (Φ_sc_ = 0.0504; P = 0.0003, F_sc_ = 0.0437; P = 0.001), suggesting genetic heterogeneity within localities. The distance tree (ML) for the CR clearly showed a reciprocally monophyly between BDT and BZ, and the WA, which is consistent with the COI results showing high bootstrap values (Fig 6) [44].

**Table 4:** Pairwise *F*_*ST*_ values (above diagonal) and *Φ*_*ST*_ values (below diagonal) for the CR, of *S*. *tiburo* from Bocas del Toro (BDT), Belize (BZ) and GeneBank sequences from the WA: North Carolina (NC), Florida bay (FB), Tampa Bay (TB), Panama City (PC).

Fst Φst	NC (n = 23)	FB (n = 25)	TB (n = 27)	PC (n = 25)	BDT (n = 15)	BZ (n = 54)
**NC**	h = 0.7194 +/- 0.0773	0.1235***	0.0427*	0.0780***	0.1754***	0.2217***
	π = 0.0010 +/- 0.0008					
**FB**	0.1743***	h = 0.9433 +/- 0.0366	0.019	-0.0062	0.0569***	0.1202***
		π = 0.0025 +/- 0.0015				
**TB**	0.1205***	0.011	h = 0.9402 +/- 0.0314	0.0019	0.0585***	0.1212***
			π = 0.0022 +/- 0.0014			
**PC**	0.0488**	0.0336*	-0.0105	h = 0.9467 +/- 0.0289	0.0552***	0.1186***
				π = 0.0027 +/- 0.002		
**BDT**	0.9334***	0.8988***	0.9060***	0.8946***	h = 0.9429 +/- 0.0404	0.1233***
					π = 0.0032 +/- 0.002	
**BZ**	0.9039**	0.8870**	0.8897**	0.8843**	0.0912**	h = 0.8246 +/- 0.0411
						π = 0.0032 +/- 0.0018

Significant P values at <0.005*

<0.002**

< 0.001***

Probability values based on 10,000 permutations. Significant P values in bold. Haplotype (h) and nucleotide (π) % ± standard deviation diversity values are shown in the diagonal for each locality. Numbers of samples of each locality are shown in parentheses.

**Table 5:** AMOVA analysis using pairwise genetic distances and conventional *F*_*ST*_ estimates.

*Φ*_*ST*_	Variance	% Total	*F*_*ST*_	P
Among groups G1 (NC,FB,TB,PC) G2 (BDT, BZ)	12.05	90.49	0.9097	0.0000+-0.0000
Among populations within groups	0.0638	0.48	0.0504	0.0003+-0.0002
Among populations	1.20	9.03	0.9049	0.2000+-0.0037
***F***_***ST***_				
Among groups G1 (NC,FB,TB,PC) G2 (BDT, BZ)	0.0214	4.37	0.0854	0.0000+-0.0000
Among populations within groups	0.0205	4.18	0.0437	0.0001+-0.0001
Among populations	0.4492	91.46	0.0437	0.3949+-0.0045

## Discussion

This study represents the first genetic analysis of the population structure of *S*. *tiburo* in the southern Caribbean. Fragments of the mitochondrial COI and CR were analyzed to evaluate the population structure and genetic diversity of fifteen *S*. *tiburo* from BDT, and the results were compared with previously published data from BZ and other locations of the WA. In spite of the high diversity of the samples analyzed, there was no evidence of genetic connectivity between the bonnethead sharks of the Caribbean (BDT and BZ) and the WA. Specifically, the significant *F*_*ST*_ and *Φ*_*ST*_ values, as well as the result of the ML trees (for both COI and CR), support the conclusion that there is significant population structure between BDT and BZ, and the WA. We found two reciprocally monophyletic clades (A and B) both in the COI and the CR genes. Based on these results, we suggest that BDT is a genetically distinct population, and add new support to the notion that there are two different lineages of bonnethead sharks (Caribbean and WA). The haplotype networks (COI and CR) also showed a clear segregation of haplotypes between BDT and BZ, and the WA, suggesting geographical isolation and limited or no gene flow between the populations analyzed.

In a previous study, Fields et al. [13] found significant population structure in the analysis of the CR from bonnetheads from the WA and BZ, which were referred to as Atlantic bonnethead (ABH) and Caribbean bonnethead (CBH) lineages, respectively. They also estimated the divergence time of the two lineages of bonnethead sharks, finding a shared a common ancestor between 3.61 and 5.2 million years before present, thus providing evidence of possible cryptic speciation [13,21,27]. Our current analyses of a more southerly sampling locality supports these two lineages and advances the case for potential speciation occurring in lower latitudes of the Western Caribbean. Therefore, our results highlight that while populations can be connected in space and time (which in theory allows random mating and gene flow), the possibility of genetic isolation and increased population structure still exists [6] as significant population structure was also detected when comparing the CR of BDT and BZ (Table 4). Currently, there are no geographic barriers that could prevent genetic interchange between the populations analyzed.

The presence of a common haplotype in COI (ST02) and one in CR (H55) between BZ and the WA, suggests that the ABH and CBH could be occurring in sympatry in BZ, indicating that the genetic connectivity between sites or hybridization could still be possible, as it has been observed in other shark species [13]. Alternatively, these shared haplotypes could be ancestral. However, despite the fact that bonnethead sharks are widely distributed along the WA, the Gulf of Mexico and the Caribbean, previous studies have suggested that this species is not highly migratory. Instead, strong site fidelity and philopatry to inshore waters could act as a mechanism for population structure of bonnethead sharks that are found in close geographical proximity [19–21,27].

Interestingly, *S*. *tiburo* female philopatry can limit the genetic connectivity between sites, as restricted home ranges and natal nursery areas have been found, even in connected geographical regions [13,19,20,23]. In BDT the population appears to be resident and closed, since all the individuals in the present study were sampled from one discrete location, despite our efforts to locate additional populations throughout the region. Anecdotal evidence from local fisherman indicates that bonnethead sharks do not occur in other areas of BDT.

Nevertheless, in this study we only included information on mitochondrial markers, which are maternally inherited and, therefore, we have no information regarding possible connectivity due to male migration. Additional analyses, including nuclear bi-parentally inherited markers should be used in order to further investigate population connectivity and potential male-mediated gene flow.

The COI haplotypes showed that all the BDT samples belonged to a single haplotype (ST01), only shared with BZ; the CR haplotype network also showed that BDT and BZ (CBH) constitute a different lineage than the populations from the WA (ABH). While the populations of the WA (ABH) appear to be in expansion corroborated by the large number of CR haplotypes (44) and the star-like topology [27], the BDT haplotypes (10) and BZ haplotypes (18) (CBH) were unique and segregated by 20 mutational steps apart from the WA the Gulf of Mexico. The CR of the BDT bonnetheads showed high nucleotide and haplotype diversity, similar to other species of sharks such as the blacktip shark, *C*. *limbatus* [45,46] and the sandbar shark, *C*. *plumbeus* [47]. This high genetic diversity is consistent with the biological characteristics of the species, defined by fast growth, early maturity, short gestation periods, and high productivity [17,20,24]. However, this is entirely speculative and would need to be validated by additional analyses from this area, and through the use of additional molecular markers.

BDT is composed of many islands, which are characterized by coral reefs, linked seagrass- mangroves ecotones, and estuaries, all of which constitute an ideal environment for bonnethead sharks, given the benefits of high prey abundance and refuge from larger elasmobranch predators. Evidence of a pattern of latitudinal variation in life history traits and reproduction of bonnethead sharks (size and age at maturation, size at birth, gestation period) has been reported for this species in closely related populations of the WA (e.g. Florida Bay and Tampa Bay) [23,24]. While entirely speculative, it is possible that these differences in life histories could be also occurring at latitudes closer to the equator, whereby relatively stable annual environmental conditions in the Caribbean could have played an important evolutionary role in shaping this CBH lineage, which could be a potential driver of cryptic speciation [23,24,48].

Our study also supports the conclusion that BDT and BZ constitute two differentiated populations, and as a primary result we provide preliminary mitochondrial evidence for BDT, to be assessed as a unique stock for management and conservation purposes. The lack of nuclear data precludes our ability to consider this area as an evolutionarily significant unit [12,49]. Complementing this study with nuclear genes could lead to a better understanding of the population structure of bonnethead sharks of BDT, and the southern Caribbean where the implications for fisheries, conservation and management should be approached carefully. Comparisons of morphometric measurements for individuals from the Caribbean and Atlantic populations could also provide additional information, needed to determine whether there are different lineages of *S*. *tiburo*. Lastly, sampling of other nearby Caribbean localities may resolve questions regarding genetic connectivity, or to find potential contact zones. Nevertheless, genetically differentiated populations should be a priority for conservation and management, since divergences in mtDNA can reflect long-term restriction of gene flow between different populations [50].

## Supporting information

S1 Table GenBank accession numbers, localities and haplotypes for the mitochondrial cytochrome oxidase I (COI) sequences. (DOCX)

S2 Table GenBank accession numbers, localities, frequencies, and haplotypes numbers for the mitochondrial control region (CR) sequences of *S*. *tiburo*. Samples from the Caribbean: Bocas del Toro (BDT) and Belize (BZ). Samples from the Western Atlantic (WA): North Carolina (NC) and three locations along the Gulf Coast of Florida: Florida Bay (FB), Tampa Bay (TB) and Panama City (PC).
(DOCX)
