# Supplementary material for: Conservation genetics of the bonnethead shark Sphyrna tiburo in Bocas del Toro, Panama: Preliminary evidence of a unique stock
Source: PLoS One. 2019 Aug 15;14(8):e0220737. doi: 10.1371/journal.pone.0220737 (PMC6695166; doi:10.1371/journal.pone.0220737)
Supplement: S1 Table — (DOCX) [file pone.0220737.s001.docx]

**Conservation genetics of the bonnethead shark *Sphyrna tiburo* in Bocas del Toro, Panama: preliminary evidence of a unique stock.**

| **Sample** | **Haplotype** | **Locality** | **Accession Number (GenBank)** |
| --- | --- | --- | --- |
| 'OC-191' | ST01 | Belize | FJ519523.1 |
| 'OC-217' | ST01 | Belize | FJ519521.1 |
| 'OC-218' | ST01 | Belize | FJ519520.1 |
| 'OC-219' | ST01 | Belize | FJ519519.1 |
| 'OC-220' | ST01 | Belize | FJ519518.1 |
| 'OC-221' | ST01 | Belize | FJ519517.1 |
| 'OC-222' | ST01 | Belize | FJ519516.1 |
| 'OC-223' | ST01 | Belize | FJ519515.1 |
| 'OC-227' | ST01 | Belize | FJ519513.1 |
| 'OC-229' | ST01 | Belize | FJ519514.1 |
| 'OC-231' | ST01 | Belize | FJ519511.1 |
| 'OC-74' | ST01 | Belize | FJ519510.1 |
| 'OC-75' | ST01 | Belize | FJ519509.1 |
| 'OC-76' | ST01 | Belize | FJ519508.1 |
| B1TCOI - B15TCOI | ST01 | Bocas del Toro | MH603121 |
| FDA100 | ST02 | Alabama | KF461242.1 |
| OC-69 | ST02 | Bagdad Beach Mexico | FJ519286.1 |
| OC-72 | ST02 | Bagdad Beach Mexico | FJ519284.1 |
| OC-198 | ST02 | Belize | FJ519522.1 |
| OC-31 | ST02 | Florida | FJ519504.1 |
| OC-32 | ST02 | Florida | FJ519503.1 |
| OC-65 | ST02 | Florida | FJ519502.1 |
| OC-255 | ST02 | Florida | FJ519501.1 |
| OC-287 | ST02 | Florida | FJ519499.1 |
| OC-259 | ST02 | Florida | FJ519498.1 |
| OC-239 | ST02 | Florida | FJ519495.1 |
| OC-240 | ST02 | Florida | FJ519494.1 |
| OC-245 | ST02 | Florida | FJ519493.1 |
| OC-246 | ST02 | Florida | FJ519492.1 |
| Stib018b(021) | ST02 | Florida Cape Canaveral | FJ519643.1 |
| Stib024b(114) | ST02 | Florida Cape Canaveral | FJ519642.1 |
| Stib016b(021) | ST02 | Florida Crooked Island Sound | FJ519644.1 |
| OC-173 | ST02 | South Carolina | FJ519491.1 |
| OC-174 | ST02 | South Carolina | FJ519490.1 |
| OC-181 | ST02 | South Carolina | FJ519489.1 |
| Stib015n(020) | ST02 | South Carolina Cape Romaine | FJ519645.1 |
| ID44 | ST02 | Texas -Gulf of Mexico | FJ519505.1 |
| OC-262 | ST02 | Florida | FJ519496.1 |
| ID42 | ST02 | Texas -Gulf of Mexico | FJ519506.1 |
| OC-230 | ST02 | Belize | FJ519512.1 |
| ID31 | ST02 | Texas -Gulf of Mexico | FJ519507.1 |
| OC-283 | ST02 | Florida | FJ519500.1 |
| OC-68 | ST02 | Bagdad Beach Mexico | FJ519287.1 |
| OC-71 | ST02 | Bagdad Beach Mexico | FJ519285.1 |
| OC-260 | ST03 | Florida | FJ519497.1 |

**S1 Table. GenBank accession numbers, localities and haplotypes for the mitochondrial Cytochrome Oxidase I (COI) sequences.**
