# Supplementary material for: Conservation genetics of the bonnethead shark Sphyrna tiburo in Bocas del Toro, Panama: Preliminary evidence of a unique stock
Source: PLoS One. 2019 Aug 15;14(8):e0220737. doi: 10.1371/journal.pone.0220737 (PMC6695166; doi:10.1371/journal.pone.0220737)
Supplement: S2 Table — Samples from the Caribbean: Bocas del Toro (BDT) and Belize (BZ). Samples from the Western Atlantic (WA): North Carolina (NC) and three locations along the Gulf Coast of Florida: Florida Bay (FB), Tampa Bay (TB) and Panama City (PC). (DOCX) [file pone.0220737.s002.docx]

| **Accession Number**  **(GenBank)** | **Haplotype** | **NC** | **FB** | **TB** | **PC** | **BDT** | **BZ** | **Total** |
| --- | --- | --- | --- | --- | --- | --- | --- | --- |
| KT031755.1 | H1 | 6 | 1 | 2 | 2 | 0 | 0 | 11 |
| KT031756.1 | H2 | 11 | 1 | 6 | 3 | 0 | 0 | 21 |
| KT031757.1 | H3 | 1 | 0 | 0 | 0 | 0 | 0 | 1 |
| KT031758.1 | H4 | 1 | 0 | 0 | 0 | 0 | 0 | 1 |
| KT031759.1 | H5 | 1 | 0 | 0 | 0 | 0 | 0 | 1 |
| KT031760.1 | H6 | 2 | 6 | 2 | 5 | 0 | 0 | 15 |
| KT031761.1 | H7 | 1 | 0 | 0 | 0 | 0 | 0 | 1 |
| KT031762.1 | H8 | 0 | 0 | 0 | 1 | 0 | 0 | 1 |
| KT031763.1 | H9 | 0 | 0 | 0 | 2 | 0 | 0 | 2 |
| KT031764.1 | H10 | 0 | 0 | 0 | 1 | 0 | 0 | 1 |
| KT031765.1 | H11 | 0 | 0 | 0 | 1 | 0 | 0 | 1 |
| KT031766.1 | H12 | 0 | 0 | 0 | 2 | 0 | 0 | 2 |
| KT031767.1 | H13 | 0 | 0 | 0 | 1 | 0 | 0 | 1 |
| KT031768.1 | H14 | 0 | 0 | 1 | 1 | 0 | 0 | 2 |
| KT031769.1 | H15 | 0 | 0 | 0 | 1 | 0 | 0 | 1 |
| KT031770.1 | H16 | 0 | 0 | 0 | 1 | 0 | 0 | 1 |
| KT031771.1 | H17 | 0 | 2 | 3 | 1 | 0 | 0 | 6 |
| KT031772.1 | H18 | 0 | 1 | 1 | 1 | 0 | 0 | 3 |
| KT031773.1 | H19 | 0 | 0 | 0 | 1 | 0 | 0 | 1 |
| KT031774.1 | H20 | 0 | 0 | 0 | 1 | 0 | 0 | 1 |
| KT031775.1 | H21 | 0 | 0 | 1 | 0 | 0 | 0 | 1 |
| KT031776.1 | H22 | 0 | 0 | 2 | 0 | 0 | 0 | 2 |
| KT031777.1 | H23 | 0 | 0 | 1 | 0 | 0 | 0 | 1 |
| KT031778.1 | H24 | 0 | 1 | 0 | 0 | 0 | 0 | 1 |
| KT031779.1 | H25 | 0 | 0 | 1 | 0 | 0 | 0 | 1 |
| KT031780.1 | H26 | 0 | 1 | 0 | 0 | 0 | 0 | 1 |
| KT031781.1 | H27 | 0 | 1 | 0 | 0 | 0 | 0 | 1 |
| KT031782.1 | H28 | 0 | 1 | 0 | 0 | 0 | 0 | 1 |
| KT031783.1 | H29 | 0 | 1 | 0 | 0 | 0 | 0 | 1 |
| KT031784.1 | H30 | 0 | 0 | 1 | 0 | 0 | 0 | 1 |
| KT031785.1 | H31 | 0 | 0 | 1 | 0 | 0 | 0 | 1 |
| KT031786.1 | H32 | 0 | 1 | 0 | 0 | 0 | 0 | 1 |
| KT031787.1 | H33 | 0 | 1 | 0 | 0 | 0 | 0 | 1 |
| KT031788.1 | H34 | 0 | 1 | 0 | 0 | 0 | 0 | 1 |
| KT031789.1 | H35 | 0 | 0 | 1 | 0 | 0 | 0 | 1 |
| KT031790.1 | H36 | 0 | 0 | 1 | 0 | 0 | 0 | 1 |
| KT031791.1 | H37 | 0 | 1 | 0 | 0 | 0 | 0 | 1 |
| KT031792.1 | H38 | 0 | 1 | 0 | 0 | 0 | 0 | 1 |
| KT031793.1 | H39 | 0 | 2 | 0 | 0 | 0 | 0 | 2 |
| KT031794.1 | H40 | 0 | 0 | 1 | 0 | 0 | 0 | 1 |
| KT031795.1 | H41 | 0 | 0 | 1 | 0 | 0 | 0 | 1 |
| KT031796.1 | H42 | 0 | 1 | 0 | 0 | 0 | 0 | 1 |
| KT031797.1 | H43 | 0 | 0 | 1 | 0 | 0 | 0 | 1 |
| KT031798.1 | H44 | 0 | 1 | 0 | 0 | 0 | 0 | 1 |
| MH603122.1 | H45 | 0 | 0 | 0 | 0 | 3 | 0 | 3 |
| MH603123.1 | H46 | 0 | 0 | 0 | 0 | 1 | 0 | 1 |
| MH603124.1 | H47 | 0 | 0 | 0 | 0 | 2 | 0 | 2 |
| MH603125.1 | H48 | 0 | 0 | 0 | 0 | 2 | 0 | 2 |
| MH603126.1 | H49 | 0 | 0 | 0 | 0 | 1 | 0 | 1 |
| MH603127.1 | H50 | 0 | 0 | 0 | 0 | 2 | 0 | 2 |
| MH603128.1 | H51 | 0 | 0 | 0 | 0 | 1 | 0 | 1 |
| MH603129.1 | H52 | 0 | 0 | 0 | 0 | 1 | 0 | 1 |
| MH603130.1 | H53 | 0 | 0 | 0 | 0 | 1 | 0 | 1 |
| MH603131.1 | H54 | 0 | 0 | 0 | 0 | 1 | 0 | 1 |
| Not Available | H55 | 0 | 0 | 0 | 0 | 0 | 2 | 2 |
| Not Available | H56 | 0 | 0 | 0 | 0 | 0 | 4 | 4 |
| Not Available | H57 | 0 | 0 | 0 | 0 | 0 | 3 | 3 |
| Not Available | H58 | 0 | 0 | 0 | 0 | 0 | 21 | 21 |
| Not Available | H59 | 0 | 0 | 0 | 0 | 0 | 8 | 8 |
| Not Available | H60 | 0 | 0 | 0 | 0 | 0 | 7 | 7 |
| Not Available | H61 | 0 | 0 | 0 | 0 | 0 | 1 | 1 |
| Not Available | H62 | 0 | 0 | 0 | 0 | 0 | 1 | 1 |
| Not Available | H63 | 0 | 0 | 0 | 0 | 0 | 1 | 1 |
| Not Available | H64 | 0 | 0 | 0 | 0 | 0 | 1 | 1 |
| Not Available | H65 | 0 | 0 | 0 | 0 | 0 | 1 | 1 |
| Not Available | H66 | 0 | 0 | 0 | 0 | 0 | 1 | 1 |
| Not Available | H67 | 0 | 0 | 0 | 0 | 0 | 1 | 1 |
| Not Available | H68 | 0 | 0 | 0 | 0 | 0 | 1 | 1 |
| Not Available | H69 | 0 | 0 | 0 | 0 | 0 | 1 | 1 |
| Not Available | H70 | 0 | 0 | 0 | 0 | 0 | 1 | 1 |
| Not Available | H71 | 0 | 0 | 0 | 0 | 0 | 1 | 1 |
| Not Available | H72 | 0 | 0 | 0 | 0 | 0 | 1 | 1 |
|  | Total | 23 | 25 | 27 | 25 | 15 | 57 | 172 |

**S2 Table. GenBank accession numbers, localities, frequencies, and haplotypes numbers for the mitochondrial Control Region (CR) sequences of *S. tiburo*.** Samples from the Caribbean: Bocas del Toro (BDT) and Belize (BZ). Samples from the Western Atlantic (WA): North Carolina (NC) and three locations along the Gulf Coast of Florida: Florida Bay (FB), Tampa Bay (TB) and Panama City (PC).
